# Effects of flickering light stimulation on retinal blood flow and full-field electroretinogram in mice

**DOI:** 10.1007/s10633-025-10049-8

**Published:** 2025-09-25

**Authors:** Milan Rai, Yamunadevi Lakshmanan, Kai Yip Choi, Henry Ho-lung Chan

**Affiliations:** 1 School of Optometry, The Hong Kong Polytechnic University, 11 Yuk Choi Road, Hung Hom, Kowloon, Hong Kong SAR, China; 2 Laboratory of Experimental Optometry (Neuroscience), School of Optometry, The Hong Kong Polytechnic University, Kowloon, Hong Kong SAR, China; 3 Centre for Eye and Vision Research (CEVR), 17W Hong Kong Science Park, Hong Kong SAR, China; 4 Research Centre for SHARP Vision (RCSV), The Hong Kong Polytechnic University, Kowloon, Hong Kong SAR, China; 5 The Hong Kong Polytechnic University, University Research Facility in Behavioral and Systems Neuroscience, Kowloon, Hong Kong SAR, China

**Keywords:** Retinal electrophysiology, Amplitudes, Electro-retinal activity, Retinal neurovascular coupling

## Abstract

**Purpose:**

To investigate the effects of brief flickering light stimulation (FLS) on retinal electrophysiology and retinal blood flow (RBF) in normal C57BL6J mice.

**Methods:**

RBF and full-field electroretinography (ffERG) were measured before and after a 60 second FLS (12 Hz, 0.1 cd·s/m^2^) in a cohort of 8-12-weeks old C57BL6J mice (*n*=10) under anaesthetic and light-adapted conditions. A separate set of age-matched mice (*n*=9) underwent RBF and ffERG measurements before and after steady light stimulation (SLS) at 1 cd/m^2^ under similar conditions. The changes in RBF (arterial and venous flow) as well as the amplitudes and implicit times of the a-wave, b-wave, oscillatory potentials (OPs), and photopic negative response (PhNR) were analyzed.

**Results:**

FLS significantly increased both arterial (*p*=0.003) and venous (*p*=0.018) blood flow as well as b-wave amplitudes (*p*=0.017) compared to SLS, which did not have any significant changes in either RBF or ERG. However, no significant differences were found in other ffERG responses (amplitudes and implicit times of a-wave, OPs, and PhNR, as well as b-wave implicit time) between the two groups after light stimulation. An increase in b-wave amplitude was positively associated with an increase in both arterial (r=0.655, *p*=0.040) and venous blood flow (r=0.638, *p*=0.047) in the FLS group.

**Conclusions:**

Our results suggest that transient FLS not only increases RBF but also enhances electro-retinal responses of the middle retinal layer, as shown by ffERG, thus demonstrating its substantial effects on both the vascular and neuronal components of retinal neurovascular coupling in mice.

**Supplementary Information:**

The online version contains supplementary material available at 10.1007/s10633-025-10049-8.

## Introduction

The robust vascular system intrinsically maintains an adequate blood supply to neural tissues, which is essential to meet the high metabolic demands associated with neural activities [1–4]. The retina, like other structures of the central nervous system, is a complex, multi-layered tissue that consists of different types of neurons, including photoreceptors, bipolar cells, and retinal ganglion cells, with high metabolic needs. These retinal neurons require large amounts of oxygen and glucose, which are supplied by the choroidal and retinal vasculature. This implies that increments in the metabolic demands of these neurons have to be supported by an augmented supply of oxygenated blood and nutrients. Retinal stimulation with flickering light has been reported to substantially increase retinal metabolism [5]. Although the metabolic demands of the outer and inner retina are similar under dark-adapted conditions, flickering light was reported to differentially affect oxygen metabolism, increasing oxygen consumption in the inner retina while decreasing it in the outer retina. A recent study on the isolated C57BL6J mouse retina, utilizing microelectrode recordings and a four-layer oxygen diffusion model, showed that outer retinal oxygen consumption under flickering light exposure was decreased to 76±14% of that observed under dark conditions, whereas inner retinal oxygen consumption increased by 6.4±5.0% [5]. Both preclinical and clinical studies have reported substantial retinal vasodilation and increased retinal blood flow (RBF) in response to high retinal metabolic activity induced by flickering light stimulation (FLS), as measured by techniques such as laser speckle flowgraphy, dual beam bi-directional Doppler Fourier-domain optical coherence tomography (OCT), and a retinal vessel analyser [6–20]. For instance, retinal stimulation with 12-Hz flickering light for 3 minutes has been shown to induce a significant increase in RBF, by more than 25% in healthy 8-week-old mice, with the response maintained longitudinally at least up to 20 weeks of age [6]. In healthy wild-type mice, FLS at 12.5 Hz (20 seconds per cycle, repeated three times with 50 seconds of baseline recording) has been found to elicit a median retinal arterial vasodilation of 2.3% (interquartile range: −1.7% to 10.0%) and a median venous vasodilation of 8.7% (Interquartile range: 5.0% to 13.2%), as assessed by area-under-the-curve analysis, which reflects the integrated vasodilatory response over time [7].

However, the retinal vascular caliber changes and increased RBF do not directly relate to the physiological status of retinal cells, as there is no direct reflection of the actual variations in retinal functional status in response to the FLS. Hence, there is limited information regarding the corresponding changes in the cellular activity (retinal function) elicited by FLS. Furthermore, the relationship between retinal vascular caliber changes and the physiological status of retinal cells remains unclear.

Therefore, this study reports the effects on retinal electrophysiological activity and RBF following a transient FLS, using full-field electroretinogram (ffERG) and Doppler spectral-domain optical coherence tomography (SD-OCT) in normal adult mice.

## Methods

### Animals

Nineteen C57BL/6J mice (age: 8-12 weeks; 10 males and 9 females), obtained from the Centralised Animal Facility of The Hong Kong Polytechnic University, were housed in a temperature-controlled room (21-22 °C) under normal lighting conditions (approximately 200 lux) with a 12/12-hour light/dark cycle. Mice were given ad libitum access to food (Pico Lab diet 20 (5053); PMI Nutrition International, Richmond, IN, USA) and water. All experimental procedures were carried out in adherence with the ARVO Statement for the Use of Animals in Ophthalmic and Vision Research. Following the completion of the experiments, the animals were housed for potential use in future research. The study protocol was approved by the Animal Ethics Sub-committee of The Hong Kong Polytechnic University (Animal Subjects Ethics Sub-committee approval number: 18-19/58-SO-R-OTHERS).

### Measurement of retinal blood flow

RBF was evaluated using an annular Doppler SD-OCT (Envisu R2210; Bioptigen, Morrisville, NC, US) as described previously [21, 22]. This OCT utilized the Doppler shift phenomenon to enable the direct visualization of flow towards and away from the objective. At first, the circular scanning with a diameter of 0.5 mm was positioned with its center located at the center of the optic nerve head. Doppler shifts appeared as red and blue signals, representing arterial (towards the objective) and venous (away from the objective) blood flow, respectively. Subsequently, ten OCT B-scan images, featuring both red and blue signals, were captured. The regions displaying Doppler shifts were then cropped using FIJI software (https://imagej.net/software/fiji/) [21, 22] for analysis. The saturation of each cropped B-scan image was adjusted using the color threshold algorithm (available in the same software under the ‘Adjust’ sub-menu of the ‘Image’ category) to include all red and blue signals in the retinal blood vessels. After removing the noise present in regions other than the blood vessels, both red and blue signals were separately quantified in terms of pixels using the ‘Measure’ function of the ‘Analyze’ category of the software. The values from ten B-scans were then averaged to determine the final red/blue pixels. The mean pixel count serves as a surrogate measure of retinal arterial (red pixels) and venous (blue pixels) calibers (per se, the blood flow), with a higher pixel count indicative of greater RBF, and a lower count indicating less RBF.

### Full-field electroretinography

The ffERG was recorded using a Ganzfeld ERG system (Q450; RETI Animal, Roland Consult, Brandenburg an der Havel, Germany). The animal, after pupil dilation, was placed on a heating pad to maintain the body temperature at around 37 °C. To prevent corneal dehydration and lens opacification due to anesthesia-induced optical changes, Lacryvisc gel (Alcon, Rueil-Malmaison, France) was applied to the corneal surface. For ffERG recording, a 2 mm-diameter gold ring electrode (Roland Consult) was placed on the cornea of each eye as an active electrode. Needle electrodes (Item No. U51-426; GVB-geliMED, Bad Segeberg, Germany) were inserted into the lateral canthus of each eye and into the upper base of the tail as reference and ground electrodes, respectively. An impedance of less than 5 kΩ was maintained for all electrodes throughout the recording period. The ERG responses were elicited by presenting a brief white LED flash (each flash duration: 4 ms) of 3.0 cd·s/m^2^ under the Ganzfeld stimulator. The signals were amplified and filtered with a bandpass of 0.1 -1000 Hz. Twenty-five ERG responses (interstimulus intervals of 1 sec) were recorded and averaged. The amplitude of the a-wave was measured from the baseline to the most negative trough (lowest point) of the a-wave, and the amplitude of the b-wave was measured from the trough of the a-wave to the peak of the b-wave. The PhNR amplitude was measured from the baseline to the negative trough of the PhNR following the b-wave. The implicit times of the a- wave, b-wave, and PhNR were measured from the onset of the stimulus to the trough of the a-wave, peak of the b-wave, and trough of the PhNR respectively. Oscillatory potentials (OPs) are high-frequency wavelets superimposed on the rising limb of the b-wave. The time domain raw data were first converted to frequency domain and then OPs were isolated through digital filtering with a bandpass of 75-300 Hz. Then, the inverse fast Fourier transform was performed to convert the filtered signal back to the time domain form. The OP amplitude was measured from the trough of the preceding wave to the peak of the wave. The implicit time of OP was measured from the stimulus onset to the peak of the wave. The amplitudes of the first four OP wavelets (labeled as OP1, OP2, OP3, and OP4) were added to present ΣOP amplitude.

### Flickering light stimulation

A custom-built device with an LED source was used to produce white flickering light . Square-wave flickering light (frequency:12 Hz, intensity: 0.1 cd·s/m^2^) was selected for retinal stimulation, as flickering light at this frequency and low intensity has been reported to effectively increase RBF in the rod-dominant retina [18, 23]. As Werkmeister et al. reported a strong increase in RBF during and after 60-second of 12 Hz FLS, the current study employed the same stimulation frequency and duration [23].

### Procedures

#### Experiment 1 – flickering light effect on retinal blood flow

**Fig. 1:**
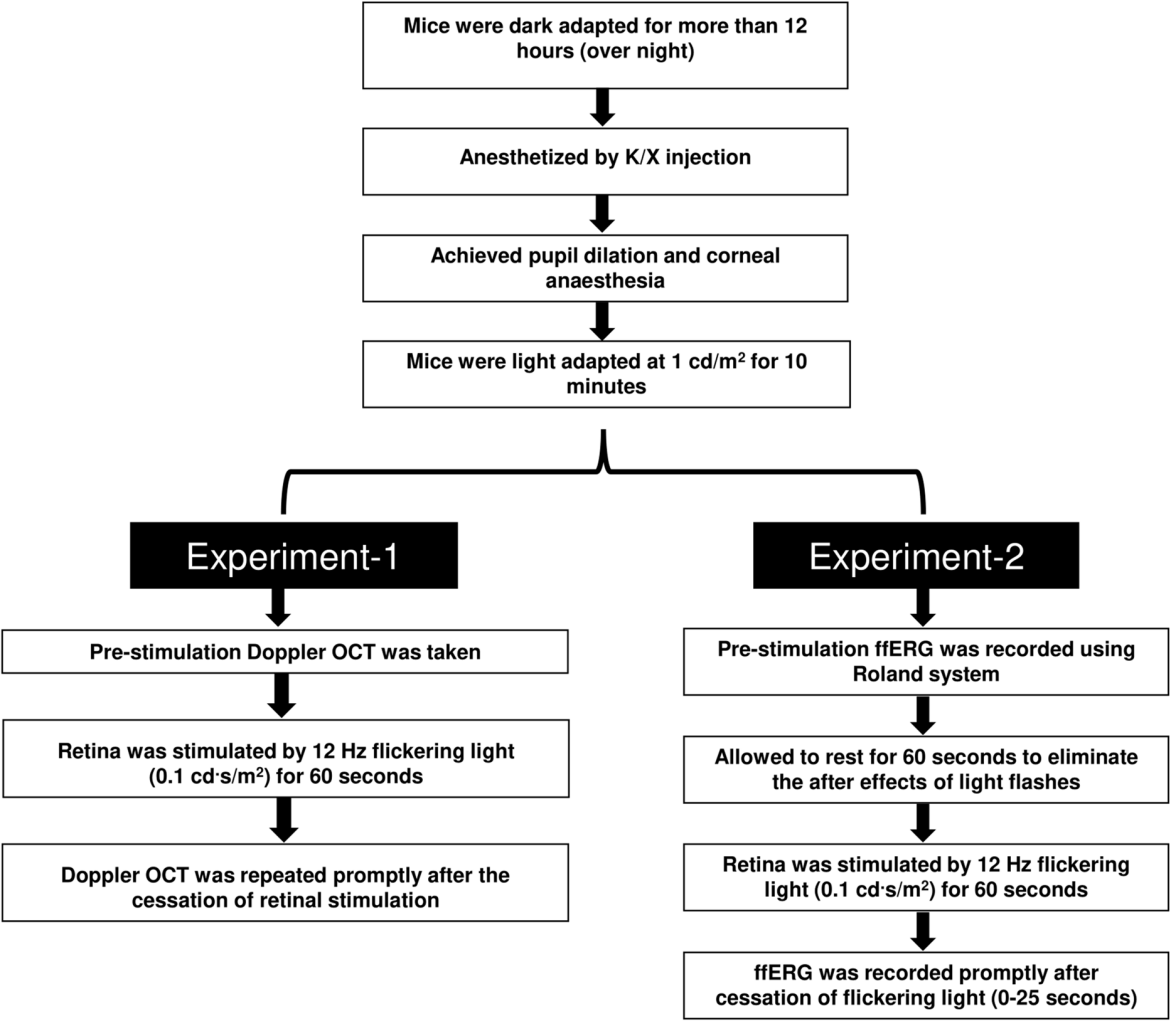
Flow diagram showing the experimental procedures of Experiments 1 and 2. Doppler OCT and ffERG recordings of light-adapted anesthetized C57BL6J mice were performed before and promptly after the cessation of 60 -second- long FLS (12 Hz, 0.1 cd·s/m^2^)

Ten mice were used to investigate the effect of FLS on RBF. Following overnight dark adaptation, animals were then anesthetized by intraperitoneal injection of a cocktail containing 90 mg/kg ketamine (Alfasan International B.V., Woerden, Holland) and 12 mg/kg xylazine (Alfasan International B.V.). Animal preparations and procedures were performed under dim red illumination. Briefly, a drop of topical anesthetics (Provain-POS 0.5% wt/vol eye drops; URSAPHARM, Saarbrücken, Germany) was applied to the eyes and the pupils were dilated by using a mydriatic agent (Mydriacyl 1% eye drops; Alcon-Couvreue, Puurs, Belgium). Lacryvisc gel (Alcon, Rueil-Malmaison, France) was applied to maintain corneal moisture throughout the experiment. One randomly selected eye was light adapted for 10 minutes (1 cd/m^2^), using the custom-built device and then baseline doppler SD-OCT was taken as described above. Subsequently, retina was stimulated using a 12 Hz flickering light of 0.1 cd·s/m^2^ for 60 seconds. Promptly after the cessation of FLS, doppler OCT was repeated. The background luminance of 1 cd/m^2^ was maintained throughout the experiment except during FLS. Figure 1 shows the experimental procedures of Experiment 1.

#### Experiment 2 – flickering light effect on retinal electrophysiological responses

One week following Experiment 1, the same cohort of ten mice was used to measure the FLS induced change in electro-retinal activity. Using the same anesthetic, mydriatic, and lubricating protocols as in Experiment 1, overnight dark-adapted mice were first light-adapted at 1 cd/m^2^ light for 10 minutes. A baseline ffERG recording was taken, and animals were then given a 60-second resting period to eliminate the aftereffects of ERG light flashes. Subsequently, the retina was exposed to the FLS for 60 seconds, and ffERG recordings were repeated promptly (within 25 seconds) after the cessation of FLS. The background luminance was constantly maintained at 1 cd/m^2^ throughout the experiment, with the exception of the 60-second FLS period. Fig 1 depicts the experimental procedures of Experiment 2.

#### Experiment 3 – steady light effect on retinal blood flow and retinal electrophysiological responses

In order to assess whether the increase in RBF and ffERG responses was specifically caused by FLS or by random variation, a control group was used. RBF and ffERG responses were obtained in a new cohort of nine mice following experimental protocols similar to Experiments 1 and 2 respectively. However, in this experiment, mice were continuously exposed to the steady light of 1 cd/m^2^ (approximately equivalent to the light energy produced by FLS of 0.1 cd·s/m^2^) for 60 seconds. The ffERG recording was carried out one week after RBF measurement to allow sufficient rest for the mice. Both procedures were conducted under red-dim illumination. A Supplementary file (ESM_1) illustrates the calculations converting the luminous intensity of the flickering light to an equivalent steady-state value.

### Statistical ANALYSIS

The ERG and RBF data from only one eye were used for statistical analyses. The data from all experiments had no outliers and followed a normal distribution (Shapiro-Wilk test, p > 0.05). Data are presented as mean ± standard error of the mean (SEM). Mixed-model ANOVA was applied to assess the difference in arterial blood flow (ABF) and venous blood flow (VBF) and ffERG waveforms (amplitudes and implicit times) between the two groups and also within the groups measured before and after the flickering or steady light stimulation with Bonferroni's pairwise post hoc comparisons. The relative changes in retinal blood flow (RBF) and ffERG amplitudes and implicit times were calculated as a percentage change from baseline. Then, an unpaired two-tailed t-test was applied to compare the difference between the FLS and SLS groups. Pearson’s correlation test was employed to determine the relationship between the percentage change in ffERGs with the percentage change in ABF and VBF. All statistical analyses were conducted using SPSS 29.0 (IBM Corp., Armonk, NY, USA). A p-value < 0.05 was considered statistically significant.

## Results

**Fig. 2:**
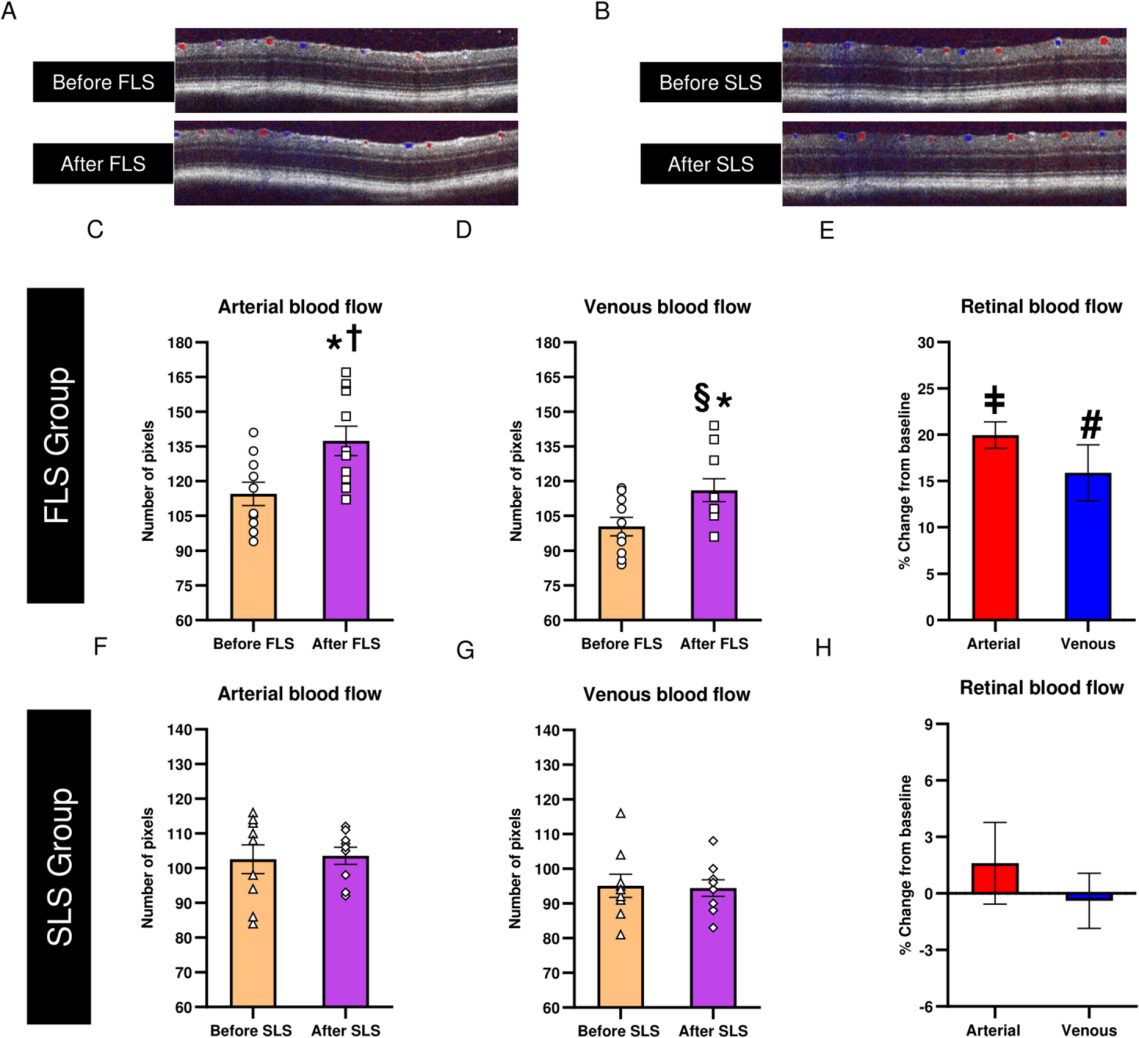
Effects of FLS and SLS on RBF, assessed by SD-OCT Doppler system. **A, B** Doppler B-scans of one representative mouse retina, from the FLS and SLS groups measured before and after stimulation. Mean values of **(C, F)** ABF and **(D, G)** VBF assessed before and after stimulation from each group and mean **(E, H)** percentage changes of ABF and VBF from the corresponding baseline from each group. Error bars represent standard error of mean (SEM). Data points represent individual mouse data. * *P* < 0.001 when compared with relative baseline, ^†^*p* < 0.001 when compared with After SLS (arterial) and, ^§^ *P* = 0.001 when compared with After SLS (venous) using mixed-model ANOVA with Bonferroni post hoc correction, ^ǂ^*P* < 0.001 and ^#^ *P* < 0.001 when compared with percentage change in ABF and VBF respectively, in the SLS group using unpaired two-tailed t-tests

Figure 2A and B show the representative Doppler B-scans (imaged before and after the light stimulations) related to RBF in the FLS and SLS groups, respectively. The mean values (in terms of pixels) of ABF (Fig. 2C, F) and VBF (Fig. 2D, G) and their corresponding percentage change (Fig. 2E, H) are also presented in Fig 2.

For both ABF and VBF, significant differences between the FLS and SLS groups were detected [Mixed-model ANOVA: (ABF: pre:post (time effect): P < 0.001, between groups: P = 0.003 and interaction effect: P < 0.001) and (VBF: time effect: P = 0.001, between groups: P = 0.018 and interaction effect: P < 0.001)]. Furthermore, RBF after FLS was significantly greater as compared with RBF after SLS (Mixed-model ANOVA with Bonferroni post hoc test: P < 0.001 for ABF and P = 0.001 for VBF) and also with pre-flickering baseline measurements (both P < 0.001). Similarly, significant differences were detected in the percentage changes in ABF and VBF between the FLS and SLS groups (Unpaired t-test: p < 0.001 for both ABF and VBF). However, SLS showed no significant changes in both ABF and VBF when compared with pre-flickering baseline measurements (P = 0.642 for ABF and P = 0.796 for VBF). All individual mean RBF data obtained from both groups are shown in the Supplementary Material (ESM_2).

**Fig. 3:**
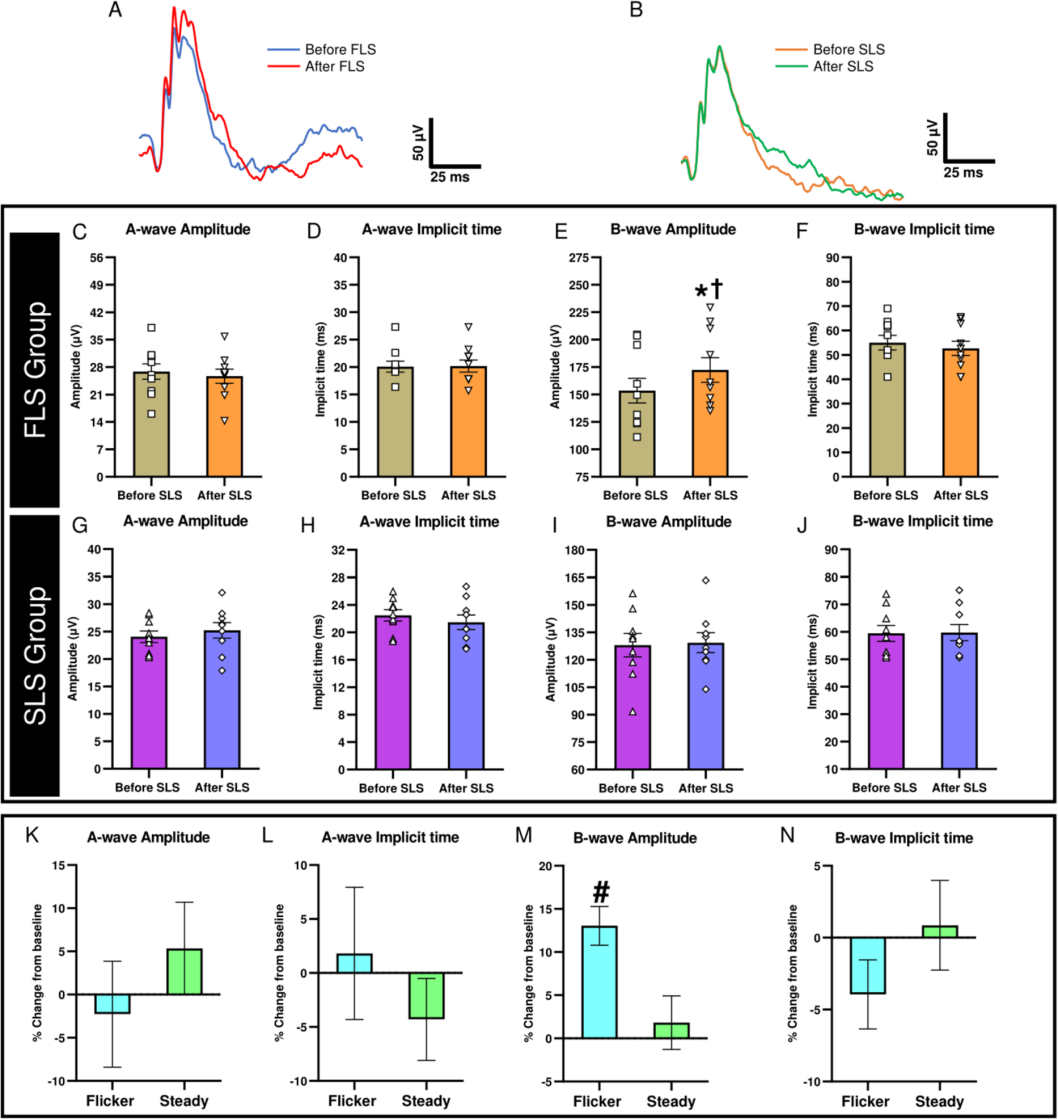
Effects of FLS and SLS on ERG a-waves and b-waves. The averaged traces of ERG waveforms of one representative mouse from **(A)** the FLS group and **(B)** the SLS group measured before and after stimulation. Mean values of ERG (**C, G**) a-wave amplitudes, **(D, H)** a-wave implicit times, (**E, I**) b-wave amplitudes, **(F, J)** b-wave implicit times and (**K, L, M, N**) the corresponding percentage changes from baseline. Each data point represents response from an individual mouse. Error bars represent SEM. * *P* < 0.001 when compared with baseline and ^†^ *P* = 0.004 when compared with b-wave amplitude measured after SLS using mixed-model ANOVA with Bonferroni post hoc correction and ^#^ *P* = 0.010 when compared with SLS group (percentage change in b-wave amplitudes from baseline) using unpaired two-tailed t-test

Fig 3A and B illustrate the traces of averaged ffERG responses recorded at two time points (before and after stimulation) from one representative animal from the FLS and SLS groups, respectively. The averaged amplitudes (Fig. 3C, E, G, I) and implicit times (Fig. 3D, F, H, J) of both a- and b-waves, measured before and after stimulation, and the corresponding percentage change (Fig. 3K, L, M, N) from baseline for both the FLS and SLS group are also presented.

The amplitude of b-wave showed a significant difference between the FLS and SLS groups as shown in Table 1. The b-wave amplitude measured after FLS was significantly greater compared to the amplitudes of b-wave recorded after SLS (post FLS vs post SLS, Mixed-model ANOVA with Bonferroni post hoc test: P = 0.004). FLS significantly increased the b-wave amplitudes as compared with its pre-flickering baseline (P < 0.001), but no significant change was observed in the SLS group (P = 0.668). There were no significant within-group temporal changes, between-group differences, or interaction effects for other ERG parameters as shown in Table 1.

**Table 1:** Mixed-model ANOVA results for ffERG responses, showing p-values for within-group pre/post (time), between-group (group), and interaction (time × group) effects.

ERG responses	Main time effect: within group (*p* -value)	Main group effect: between groups (*p* -value)	Interaction effect (*p* -value)
A-wave amplitude	0.991	0.424	0.348
A-wave implicit time	0.567	0.133	0.495
**B-wave amplitude**	**< 0.001**	**0.017**	**< 0.001**
B-wave implicit time	0.389	0.168	0.287
PhNR amplitude	0.308	0.658	0.589
PhNR implicit time	0.114	0.318	0.913
OP1 amplitude	0.118	0.615	0.772
OP1 implicit time	0.119	0.097	0.477
OP2 amplitude	0.227	0.675	0.928
OP2 implicit time	0.707	0.089	0.196
OP3 amplitude	0.103	0.943	0.192
OP3 implicit time	0.918	0.393	0.207
OP4 amplitude	0.180	0.609	0.443
OP4 implicit time	0.767	0.647	0.991
ΣOPs amplitude	0.073	0.739	0.820

**Fig. 4:**
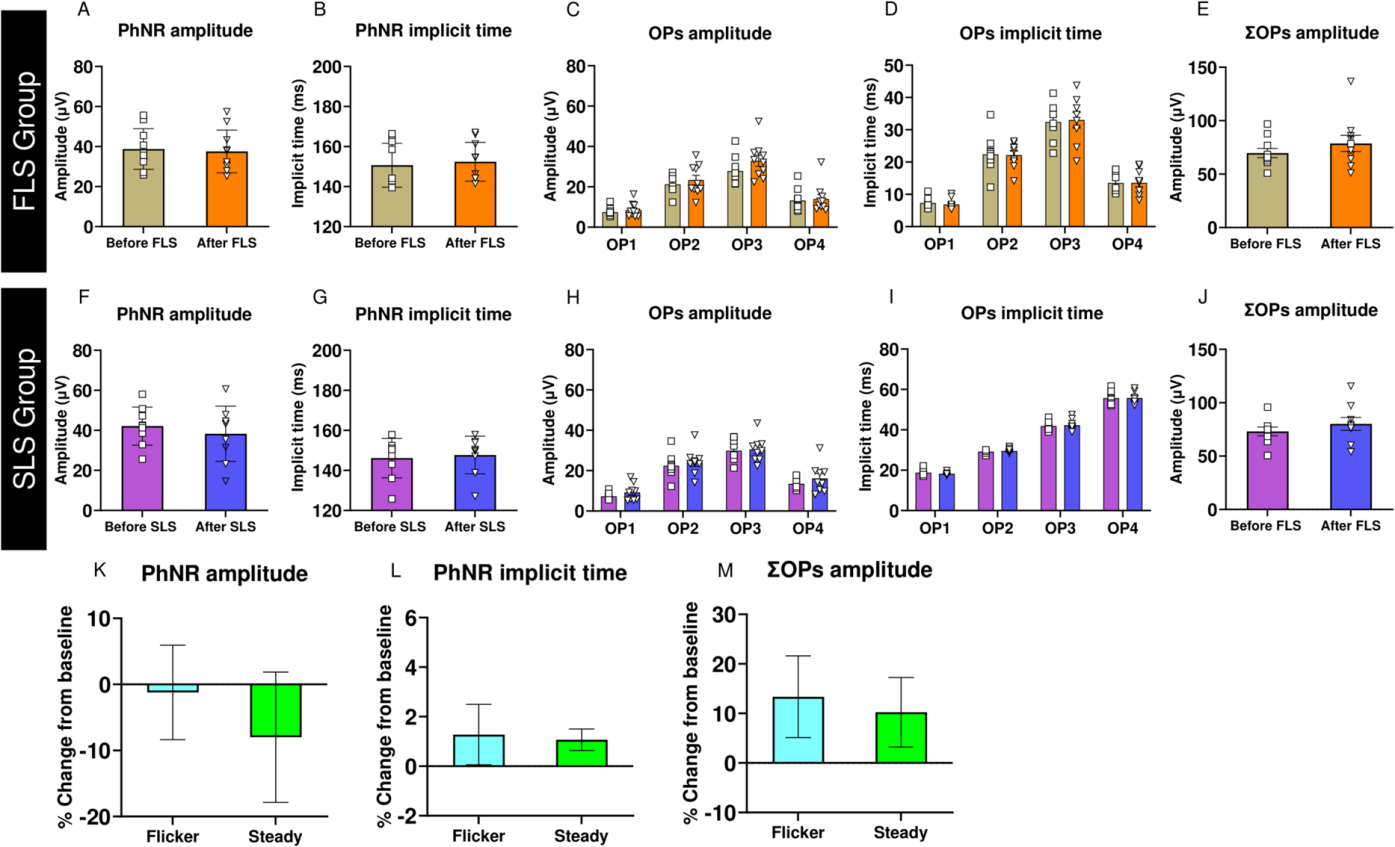
Effects of FLS and SLS on OPs and PhNR. Mean values of (**A, F**) PhNR amplitudes, **(B, G)** PhNR implicit times, (**C, H**) OPs amplitudes, **(D, I)** OPs implicit times, (**E, J**) summed OPs amplitudes measured before and after light stimulation. Mean percentage changes in **(K)** PhNR amplitudes, **(L)** PhNR implicit times, and **(M)** summed OPs amplitudes from corresponding baseline. Each symbol represents the response of a single mouse. Error bars denote SEM.

Fig 4 presents the mean amplitudes and implicit times of the PhNR and individual OPs (OP1, OP2, OP3, and OP4), as well as the summed amplitudes of OPs (ΣOPs), for both experimental groups. In addition, the mean percentage changes in PhNR amplitude, PhNR implicit time, and ΣOPs amplitude relative to baseline are shown in Fig 4K-M, respectively. Bold indicates significant effects.

The percentage change in b-wave amplitude was also significantly greater in FLS as compared with the SLS group (Unpaired t-test: P = 0.010). However, no significant differences were found in percentage change in b-wave implicit times (Unpaired t-test: P = 0.241), a-wave amplitudes (Unpaired t-test: P = 0.362), a-wave implicit times (Unpaired t-test: P = 0.410), PhNR amplitudes (Unpaired t-test: P = 0.586), PhNR implicit times (Unpaired t-test: P = 0.878), and ΣOPs amplitudes (Unpaired t-test: P = 0.778), between the two groups.

**Fig. 5:**
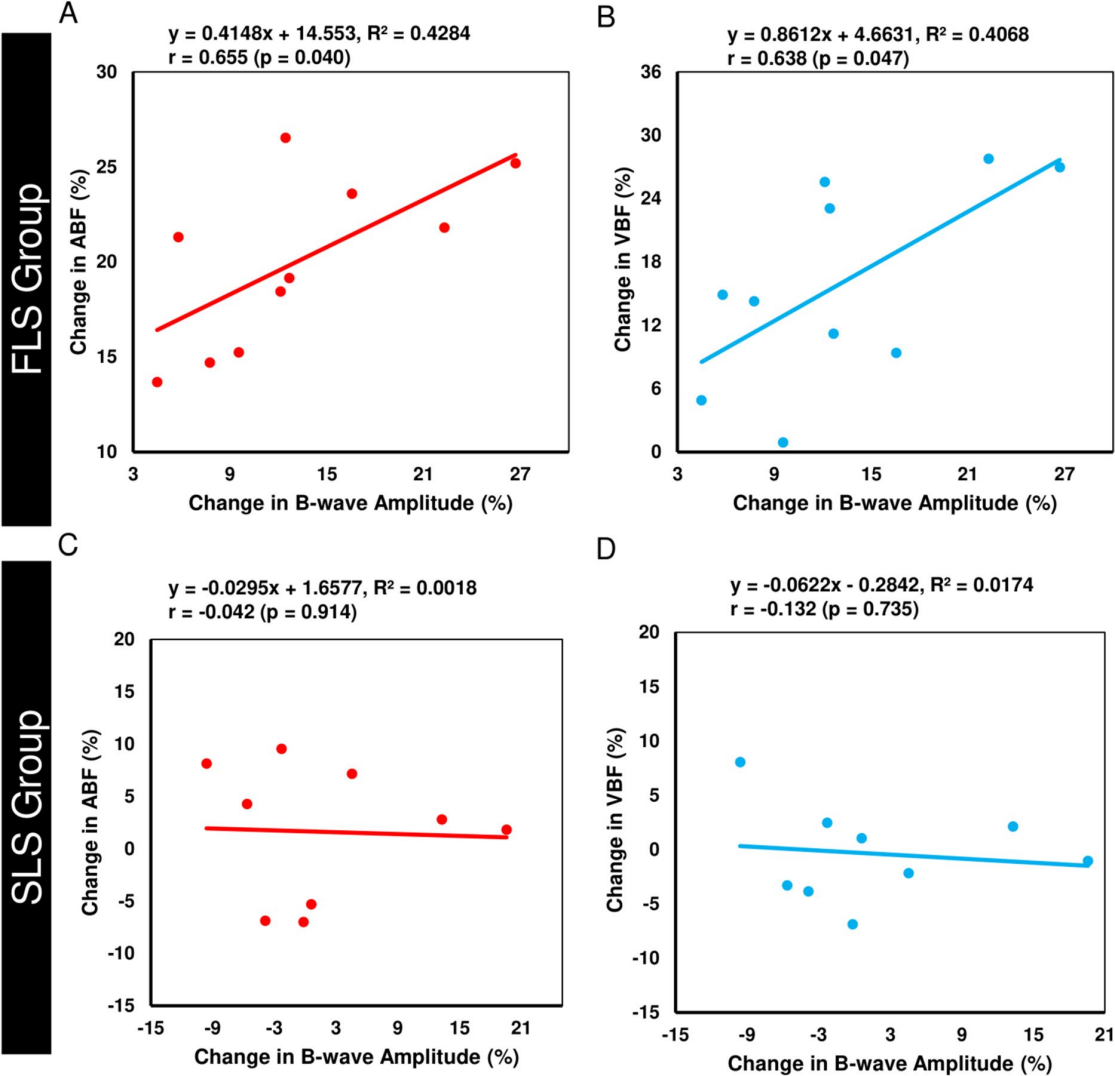
Correlations of percentage change in b-wave amplitude with percentage change in ABF and percentage change in VBF in the FLS (**A** and **B**) and SLS (**C** and **D**) groups.

Notably, a significant positive correlation was found between the percentage change in b-wave amplitudes and percentage change in ABF (r = 0.655, P = 0.040) and VBF (r = 0.638, P = 0.047) in the FLS group but not in the SLS group (ABF: r =  −0.042, P = 0.914; VBF: r =  −0.132, P = 0.735) (Fig. 5) Also, no significant correlations were found between the percentage changes in other ERG responses and the percentage changes in ABF or VBF in either the FLS or SLS groups as shown in Table 2.

**Table 2.** Correlation analysis between percentage changes in ERG responses and RBF in the FLS and SLS groups

ERG measures (%Δ)	%Δ ABF				%Δ VBF			
	FLS group		SLS group		FLS group		SLS group	
	r-value	*p*-value	r-value	*p*-value	r-value	*p*-value	r-value	*p*-value
%Δ a-wave amplitude	−0.342	0.333	0.072	0.853	0.257	0.474	0.022	0.955
%Δ a-wave implicit time	−0.142	0.695	0.402	0.284	0.175	0.628	0.539	0.135
%Δ b-wave amplitude	**0.655**	**0.040**	−0.042	0.914	**0.638**	**0.047**	−0.132	0.735
%Δ b-wave implicit time	0.338	0.339	0.476	0.195	0.223	0.536	0.334	0.380
%Δ PhNR amplitude	−0.334	0.346	0.259	0.501	−0.496	0.145	0.312	0.414
%Δ PhNR implicit time	−0.08	0.827	-0.039	0.921	−0.423	0.224	−0.154	0.693
%Δ ΣOPs amplitude	−0.121	0.739	0.196	0.613	0.072	0.844	0.297	0.440

Table 2. Correlation analysis between percentage changes in ERG responses and RBF in the FLS and SLS groups. Bold indicates significant differences. Supplementary Material (ESM_3) provides all individual mean ffERG data obtained from both groups.

## Discussion

To our knowledge, the present study is the first to report the effects of FLS on electrical activities together with blood flow change in the mouse retina. The findings suggested that the FLS increased the electrophysiological responses, originating primarily from the mid-retinal layer (i.e., bipolar cells, mainly represented by b-waves) and the RBF. Previous studies largely reported the effects of flickering light on RBF velocity and/or blood volume [6–20]. However, no study has yet reported the changes in retinal electrophysiological responses promptly after FLS. Therefore, the current study employed ERG, in addition to RBF measurement, to investigate the flicker-induced neuronal activities in the retina, thus reporting the changes in both vascular and neuronal components.

In this study, the average increase in ABF and VBF was 19.96±1.41% and 15.89±3.02 % respectively. A previous study using C57BL6J mice reported a maximum increase of 32.5 ± 5.0% in RBF during 3 minutes of retinal stimulation (12 Hz, 30 lux) [18]. Another study detected an increase of 22.79-26.37% in relative RBF during 20 seconds of FLS (10 Hz, 1200 lux) using a similar mouse strain [24]. It is apparent that the increase in RBF varied between studies and this could be attributed to the differences in the measurements used (laser speckle flowgraphy vs Doppler OCT), stimulus parameters (such as duration, frequency, luminance/intensity of flickering light) and methodology (during vs after stimulation) employed. Our results showed that the b-wave amplitude measured promptly after FLS was significantly increased by 13.04±2.23%, although post-flickering b-wave implicit time did not differ significantly from the corresponding baseline value. On exposure to SLS using a similar light energy-matched luminance, the post-stimulus b-wave amplitudes were similar to the corresponding baseline. These suggested that the FLS (in terms of its temporal characteristics) induced significant effects on the retina by increasing the RBF and ERG amplitude as compared with SLS. The neuro-associated vascular changes observed in this study may be related to the neurovascular coupling, which is an auto-regulatory mechanism linking the transient increase in metabolic demands of local neural tissues with subsequent augmented blood supply to those tissues [1]. Previous studies have reported that the retinal stimulation by flickering light induces neurovascular coupling-related activities whereby retinal vasodilation occurs, increasing the retinal blood supply to sustain the high metabolic demands of retinal cells caused by the repeated light stimulation [6–20]. The augmented blood supply nourishes the retinal cells by providing additional amounts of nutrients, oxygen and electrolytes, thereby supporting the physiological responses of retinal neurons, which is believed to be reflected by the electro-retinal activities measured by ERG in response to FLS.

Our findings revealed that the post-flickering RBF and b-wave amplitudes were significantly greater than the pre-flickering values. Surprisingly, flicker-induced change in RBF was positively associated with the change in b-wave amplitudes. One possible explanation for these findings is that the ketamine-xylazine cocktail reduced mean arterial blood pressure (most likely RBF as well) and blood oxygenation during anesthesia, leading to a subnormal metabolic and hypoxic conditions in the retina [25, 26]. This likely affected the physiological responses of the middle retinal cells, resulting in smaller b-wave amplitudes at the pre-flickering condition. However, the imposition of flickering light significantly increased RBF, which provided additional nutrients and oxygen to the retina and improved its metabolic activity. This supported the physiological responses of middle retinal layer and enhanced the b-wave amplitudes. In contrast, steady light stimulation did not increase RBF and, therefore, could not enhance b-wave amplitudes. Furthermore, our findings are also supported by previous studies which reported a rapid loss of ERG b-wave in hypoxemic [27], hypoglycemic [28], and ischemic conditions [29], indicating the critical role of adequate blood supply in sustaining normal retinal physiology. However, since the a-wave is more resistant to hypoxemia and ischemic conditions, no significant changes in a-waves were obtained after FLS [27, 30]. Furthermore, while the amplitudes of OPs showed a tendency to increase after FLS, this did not reach statistical significance. A similar increasing trend was also observed in the SLS group, suggesting that the responses of the inner retinal inhibitory circuitry were not significantly altered by FLS. Moreover, the functional responses of the RGCs, as reflected by the PhNR, showed no significant variation under any light stimulation condition. The concept of coupling between augmented blood flow and neuronal metabolic demands is further supported by our findings and by *in vivo* RBF measurements in rats reported by Kornfield et al. [12]. Their study demonstrated that blood flow in the capillaries of the superficial vascular layer in the retina, which supplies blood to the RGCs, increased by less than 1% during 15 seconds of FLS, indicating no significant alteration in metabolic demands in the innermost retinal layer during FLS. However, the blood flow in the capillaries of the intermediate vascular layer, which supplies the middle retinal layer including bipolar cells, increased by more than 25% during FLS. Furthermore, FLS induced more than 10% increase in blood flow in the capillaries of the deep vascular layer, which partially supplies the middle retinal layer [12]. Although capillary-level blood flow changes in the three vascular layers were not directly assessed in our study, FLS might have induced a substantial increase in the blood supply within the capillaries of the middle retinal layer, similar to what was observed in the rat retina [12]. This increased blood supply might have contributed to the significant enhancement of functional responses of the middle retinal layer as shown by enhanced b-wave amplitudes after a 60-second FLS in the present study. These results suggest that the physiological mechanism of flicker-induced augmented blood flow to the retina is mainly related to the increased inner retinal metabolism, as inner retinal neurons undergo net depolarization and increased firing rate during flickering light exposure [5]. Buerk et al. reported an increase in K^+^ production correlated with the blood flow changes at the surface of the optic nerve head in response to FLS [31]. Dmitriev et al. recently reported a sustained increase in K^+^ concentration in the inner retina, when an isolated mouse retina was stimulated with flickering light, which suggested increased metabolic demands in that region [32]. The continuous transport of K^+^ requires an additional supply of adenosine triphosphate (ATP) molecules to function against the concentration gradient. An increase in RBF is believed to meet the augmented metabolic demand caused by high demand of ATP, which may, in turn support the physiological activity of the retinal cells. Supporting this, Kornfield and co-workers reported the maximum increase in RBF within the intermediate and deep vascular layers, which supply the terminals of bipolar cells [10]. Notably, our study demonstrated that FLS on the retina led to a significant increase in electrophysiological responses, predominantly from the mid-retinal region, which is likely due to the retinal bipolar cells.

One of the limitations of this study is that the ketamine-xylazine cocktail was used as a general anesthetic, which has been reported to affect the hemodynamic conditions by lowering systemic blood pressure in mice [25]. Moreover, mice anesthetized with injectable and inhalant anesthesia are prone to hypoxia [26]. Therefore, further investigations are required to assess the flicker-induced changes in RBF and ERG responses under conditions of supplemental oxygen administration in anesthetized mice. Nonetheless, even under the influence of ketamine-xylazine, Tamplin et al., using the same mouse strain (C57/BL6J) as the present study, also reported a significant increase in RBF compared to baseline during flickering light exposure [24]. Another limitation of this study is the distinct application of low-intensity flicker settings (Experiment 1 and 2) and low-luminance steady-state conditions (Experiment 3). The intended luminance level for the SLS was 1.2 cd/m^2^ to match the luminous energy produced by 0.1 cd·s/m^2^. However, due to technical limitations of the employed device, a luminance level of 1.0 cd/m^2^ was utilized. However, this distinction, despite precluding direct comparability between the two values, entails that both the flickering and steady-state conditions utilized low-level stimuli. This could have had a very mild impact on the findings of our study. As previous studies have employed a wide range of flickering light parameters [6–20], future studies should investigate the effects of varying temporal frequencies, duration of stimulation, light intensity, and wavelength on ERG parameters as well as on RBF responses. Findings from such studies would provide a comprehensive evaluation of how different light parameters modulate the functional and hemodynamic responses.

By applying our experimental protocols (both RBF and ERG), further studies can be carried out to explore neuro-associated vascular changes that mimic ocular conditions related to retinal vasculature, such as hypertensive and diabetic retinopathies. Comprehending the relationship between the retinal neural and vascular component through applications of ERG and RBF, respectively, could facilitate a deeper understanding of how the neural activity and vascular responses are coupled under the influence of flickering light stimuli. This may initiate potential applications in clinical practice.

In conclusion, this study quantified the changes in electro-retinal responses in response to FLS and demonstrated an association between flicker-induced changes in RBF and ERG responses in the wild type normal mice. The increased retinal electrophysiological response from the mid-retinal layer following a brief FLS is positively correlated with the increase in RBF, which was possibly related to the effect of retinal neurovascular coupling.

## Supplementary Information

Below is the link to the electronic supplementary material.

Supplementary file1 (PDF 9 KB)

Supplementary file2 (XLSX 14 KB)

Supplementary file3 (XLSX 23 KB)

## Declarations

### Statement on the welfare of animals

All applicable international, national and/or institutional guidelines for the care and use of animals were followed. All procedures performed in studies involving animals were in accordance with the ethical standards of the institution or practice at which the studies were conducted.
