# Supplementary material for: Effects of flickering light stimulation on retinal blood flow and full-field electroretinogram in mice
Source: Doc Ophthalmol. 2025 Sep 25;151(3):205–18. doi: 10.1007/s10633-025-10049-8 (PMC12568800; doi:10.1007/s10633-025-10049-8)
Supplement: Supplementary file 1 — Supplementary file1 (PDF 9 KB) [file 10633_2025_10049_MOESM1_ESM.pdf]

Effects of flickering light stimulation on retinal blood flow and full-field electroretinogram in mice; Documenta Ophthalmologica; Milan Rai, Yamunadevi Lakshmanan, Kai Yip Choi, Henry Ho-lung Chan. Corresponding author: Henry Ho-lung Chan; The Hong Kong Polytechnic University, School of Optometry, Kowloon, Hong Kong SAR, China, The Hong Kong Polytechnic University, School of Optometry, Laboratory of Experimental Optometry (Neuroscience), Kowloon, Hong Kong SAR, China, Centre for Eye and Vision Research (CEVR), 17W Hong Kong Science Park, Hong Kong SAR, China, The Hong Kong Polytechnic University, Research Centre for SHARP Vision (RCSV), Kowloon, Hong Kong SAR, China, The Hong Kong Polytechnic University, University Research Facility in Behavioral and Systems Neuroscience, Kowloon, Hong Kong SAR, China; henryhl.chan@polyu.edu.hk

Supplementary information

For Flickering light stimulation, following flickering light parameters were used:

Frequency: 12 Hz

Intensity of each flash: 0.1 cd·s/m^2

Duration of FLS: 60 seconds

Total number of flicker flashes in 60 seconds: 12 X 60 = 720

Total luminous exposure over 60 seconds: 720 X 0.1 cd·s/m^2

Now, to find the equivalent steady luminance, this total luminous exposure should be evenly distributed over the 60-second duration.

Hence, Equivalent steady luminance = Total luminous exposure / Duration

Equivalent steady luminance = 720 X 0.1 cd·s/m^2 / 60 s

Equivalent steady luminance = 1.2 cd/m^2

Therefore, steady light luminance of 1.2 cd/m^2 should be used constantly for 60 seconds to have the same amount of light energy received per unit area
